# Metabolic Dysfunction–Associated Steatotic Liver Disease: From Pathogenesis to Current Therapeutic Options

**DOI:** 10.3390/ijms25115640

**Published:** 2024-05-22

**Authors:** Piero Portincasa, Mohamad Khalil, Laura Mahdi, Valeria Perniola, Valeria Idone, Annarita Graziani, Gyorgy Baffy, Agostino Di Ciaula

**Affiliations:** 1 Clinica Medica “A. Murri”, Department of Precision and Regenerative Medicine and Ionian Area (DiMePre-J), University of Bari “Aldo Moro”, 70124 Bari, Italy; mohamad.khalil@uniba.it (M.K.); lauramahdi5@gmail.com (L.M.); valeriaperniola17@gmail.com (V.P.); vidone@aboca.it (V.I.); agostinodiciaula@tiscali.it (A.D.C.); 2 Aboca S.p.a. Società Agricola, 52037 Sansepolcro, Italy; 3 Institut AllergoSan Pharmazeutische Produkte Forschungs- und Vertriebs GmbH, 8055 Graz, Austria; graziani@allergosan.at; 4 Division of Gastroenterology, Hepatology and Endoscopy, Department of Medicine, Brigham and Women’s Hospital, Harvard Medical School, Boston, MA 02115, USA; gbaffy@mgb.org; 5 Section of Gastroenterology, Department of Medicine, VA Boston Healthcare System, Boston, MA 02132, USA

**Keywords:** clinical trials, drug therapy, fatty liver, liver fibrosis, MAFLD, MASLD, NAFLD, NASH

## Abstract

The epidemiological burden of liver steatosis associated with metabolic diseases is continuously growing worldwide and in all age classes. This condition generates possible progression of liver damage (i.e., inflammation, fibrosis, cirrhosis, hepatocellular carcinoma) but also independently increases the risk of cardio-metabolic diseases and cancer. In recent years, the terminological evolution from “nonalcoholic fatty liver disease” (NAFLD) to “metabolic dysfunction-associated fatty liver disease” (MAFLD) and, finally, “metabolic dysfunction-associated steatotic liver disease” (MASLD) has been paralleled by increased knowledge of mechanisms linking local (i.e., hepatic) and systemic pathogenic pathways. As a consequence, the need for an appropriate classification of individual phenotypes has been oriented to the investigation of innovative therapeutic tools. Besides the well-known role for lifestyle change, a number of pharmacological approaches have been explored, ranging from antidiabetic drugs to agonists acting on the gut–liver axis and at a systemic level (mainly farnesoid X receptor (FXR) agonists, PPAR agonists, thyroid hormone receptor agonists), anti-fibrotic and anti-inflammatory agents. The intrinsically complex pathophysiological history of MASLD makes the selection of a single effective treatment a major challenge, so far. In this evolving scenario, the cooperation between different stakeholders (including subjects at risk, health professionals, and pharmaceutical industries) could significantly improve the management of disease and the implementation of primary and secondary prevention measures. The high healthcare burden associated with MASLD makes the search for new, effective, and safe drugs a major pressing need, together with an accurate characterization of individual phenotypes. Recent and promising advances indicate that we may soon enter the era of precise and personalized therapy for MASLD/MASH.

## 1. Introduction

The epidemiological progression of liver steatosis is closely linked to the steady increase in overweight and obesity, adding to a huge social, economic, and healthcare burden globally [1,2]. In fact, the prevalence of obesity has dramatically increased over the past 30–40 years in adults [3,4], children and adolescents [5]. Overweight and obesity, in turn, increase the risk of type 2 diabetes mellitus (T2DM), arterial hypertension, atherosclerosis, cardiovascular diseases [6,7], metabolic syndrome, dyslipidemia, cholesterol cholelithiasis [8], several malignancies [9], increased risk of severe COVID-19 [10], and metabolic dysfunction–associated steatotic liver disease (MASLD), among many other conditions and related complications.

**Figure 1:**
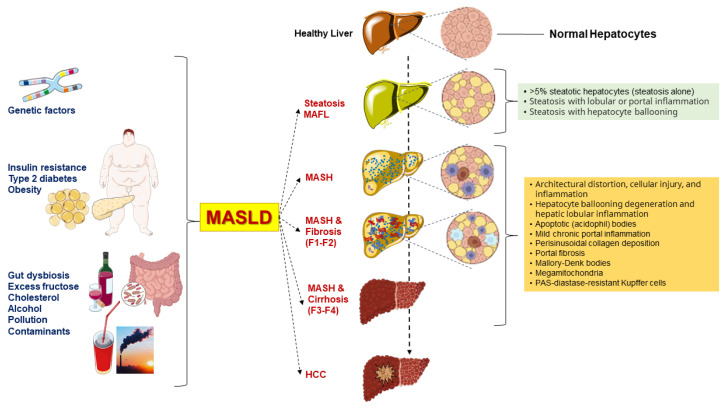
Genetic background and various metabolic dysfunctions contribute to the advancement of metabolic dysfunction-associated steatotic liver disease (MASLD) [17]. Besides the genetic predisposition, several metabolic dysfunctions, including visceral obesity and type 2 diabetes mellitus (T2DM), are primary risk factors for MASLD progression. Other factors can also contribute to the environmental background and include gut dysbiosis, excess dietary fructose, cholesterol, alcohol intake, environmental pollution, and food contaminants [17,18]. MASLD is characterized by intrahepatic triglyceride accumulation exceeding 5% and follows a complex continuum spectrum of disease [19]. In MAFL, the picture is characterized by steatosis alone, portal inflammation, or hepatocyte ballooning. In MASH, the typical findings include architectural distortion, cellular injury, and inflammation, hepatocyte ballooning degeneration and hepatic lobular inflammation, acidophil apoptotic bodies, mild chronic portal inflammation, perisinusoidal collagen deposition resulting in zone 3 accentuation in a “chicken wire” pattern, portal fibrosis without perisinusoidal or pericellular fibrosis, Mallory-Denk bodies (previously called Mallory bodies or Mallory hyaline), mega-mitochondria, PAS-diastase-resistant Kupffer cells, glycogenated (vacuolated) nuclei in periportal hepatocytes, lobular lipogranulomas, mild hepatic siderosis involving periportal hepatocytes or panacinar reticuloendothelial cells, and macronodular cirrhosis, which is an end-stage result of MASH [15]. About 22% of individuals progress from MASH to cirrhosis, and those with severe cirrhosis may develop hepatocellular carcinoma (HCC). F1: portal fibrosis without septa. F2: portal fibrosis with few septa. F3: numerous septa without cirrhosis. F4: cirrhosis.

The first definition of liver disease associated with metabolic dysfunction dates back to 1980 when the definition “nonalcoholic steatohepatitis” (NASH) was coined to identify a group of histological features reminiscent of alcohol-associated liver injury among non-drinker individuals [11]. In 1986, nonalcoholic fatty liver disease (NAFLD) was introduced as a term to describe the histological presence of steatosis in at least 5% of hepatocytes [12]. Later, NAFLD was defined as >5.5% liver fat content by magnetic resonance proton spectroscopy in individuals with no or little alcohol consumption (i.e., a daily alcohol intake of ≤20 g in females and ≤30 g in males) [13]. The definition of NAFLD and NASH required the exclusion of other, well-defined causes of chronic liver disease such as viral hepatitis, genetically determined liver diseases (e.g., Wilson disease), and drug-induced or toxic liver injury [11,14,15]. The acronym NAFLD includes a spectrum of hepatic disorders ranging from macrovesicular steatosis (NAFL) with or without mild lobular inflammation [16], to NASH, with hepatocellular injury (ballooning), inflammation, and perisinusoidal fibrosis. Further progress to advanced fibrosis, cirrhosis, and hepatocellular carcinoma is also possible (Figure 1).

In the last 3 years, two panels of scholars from mostly different geographical regions of the world have advocated a change of terminology to move from NAFLD, a diagnosis of exclusion, to more pro-active and metabolically-oriented diagnostic terms. Thus, “metabolic dysfunction-associated fatty liver disease” (MAFLD) was proposed in 2020 [20,21], and “metabolic dysfunction-associated steatotic liver disease” (MASLD) and “metabolic dysfunction-associated steatohepatitis” (MASH) were proposed in 2023 [22]. These changes in the nomenclature of the most commonly observed chronic liver disease, which has been known as NAFLD for the past 40 years, intend to propose a new framework for researchers, practitioners, and patients. The introduction of the acronym MASLD has been made within the construct of steatotic liver disease (SLD) as an umbrella term that facilitates the classification of various liver disorders with abnormal fat accumulation [22]. In addition, with MAFLD removing the potentially stigmatizing term “[non]alcoholic” followed by the purging of “fatty” from the term MASLD, the negative impact of two potentially stigmatizing terms on public perception has now been overcome [22]. 

The rationale behind these changes has been to improve disease classification, increase disease awareness, and emphasize the link between cardiometabolic risk and all-cause mortality [23]. Epidemiological evidence shows that there is an almost complete overlap of populations when using the terminology MASLD with respect to NAFLD, i.e., ~99% of individuals with NAFLD meet MASLD criteria [24,25,26]. However, it is expected that the latest consensus on the nomenclature change will facilitate the development of personalized therapeutic approaches [27,28]. Furthermore, emerging evidence indicates that the novel criteria for the classification of subjects with steatosis according to clinical phenotype will allow better identification of patients with normal weight and steatosis [29].

We will use in the following paragraphs the terms MASLD and MASH, which have de facto replaced prior terms. However, we are aware that current evidence may not be strong enough to convincingly allow a full translation of data obtained on NAFLD in the last decades, and on MAFLD in the last 4 years, as they pertain to the newest disease definition. We briefly discuss the main clinical and pathogenetic aspects of MASLD. We also discuss the most important and recent advances of pharmacotherapy proposed in the management of subjects with fat over-storage in the liver. In this respect, the terminological change from NAFLD to MAFLD and MASLD has been paralleled, in the last decade, by significant advancement in the knowledge of mechanisms linking the steatotic liver with systemic pathogenic pathways leading to increased cardio-metabolic risk. This, in turn, has generated a number of pharmacological approaches ranging from antidiabetic drugs to agonists acting on the gut–liver axis and at a systemic level (mainly farnesoid X receptor [FXR] agonists, PPAR agonists, thyroid hormone receptor agonists), anti-fibrotic and anti-inflammatory agents.

## 2. Clinical Manifestations

The clinical course of MASLD is highly variable and poorly predictable, although potentially lethal. The majority of individuals with MASLD will remain asymptomatic and this condition can last until decompensated cirrhosis develops [30]. Early clinical symptoms, if occur, are typically mild and include right upper quadrant pain and fatigue. Usually, ultrasonography reveals a “brighter” steatotic liver, as compared with the right kidney cortex [15]. The overall impression of the sonographer for the presence of macrovesicular hepatic steatosis of any degree has a sensitivity of 60.9% and a specificity of 100%. The sensitivity and specificity increase to 100% and 90% respectively, only if >20% of fat over-storage is present in the liver [31]. We recently showed by an artificial-intelligence-related algorithm, that ultrasonography becomes a reliable method for the evaluation and classification of liver steatosis. In fact, this imaging technique generates acceptable outcomes at least in subjects with liver fat percentage above 7.2%, i.e., very close to the accuracy of nuclear magnetic resonance [32].

Increased levels of serum alanine aminotransferase (ALT) and aspartate aminotransferase (AST) point to hepatocellular injury [33] but they can fluctuate over time [15], and might not address the presence of fibrosis [33]. The simple fatty liver (MASL) can be diagnosed non-invasively by imaging. However, a liver biopsy is still necessary to diagnose MASH, a phenotype characterized by the presence of hepatocyte ballooning, lobular inflammation, macrovesicular steatosis, and, very frequently, perisinusoidal fibrosis [15]. These histological features contribute to the construction of the MASLD activity score (NAS), especially useful for quantifying changes during therapeutic trials [34]. A NAS of ≥5 correlates highly with a diagnosis of MASH [35].

In a meta-analysis, rates of fibrosis progression were slower in patients with simple steatosis compared to patients with MASH, i.e., 1 stage of progression over 14.3 years and 7.1 years, respectively [16]. Nevertheless, about one-fifth of patients with MASH rapidly progress towards more aggressive forms of steatohepatitis with significant fibrosis. This progression is promoted by environmental and genetic factors, which remain largely unknown [17,36]. In particular, about 60% of MASLD might progress to MASH featuring inflammatory infiltration and significant fibrosis [37]. From this point, about 22% of patients with MASH-related fibrosis will progress to cirrhosis, and about 2% might progress to hepatocellular carcinoma [19,37].

In addition, MASLD per se puts the patients at risk for several extrahepatic complications, including cardiovascular disease (CVD) and T2DM. Indeed, patients with severe liver fibrosis likely develop subclinical carotid atherosclerosis with cardiovascular diseases, which account for the majority of MASLD-related mortality [38]. Additional extrahepatic diseases include chronic kidney disease, and a few types of extrahepatic cancers [39,40]. Given that the global prevalence of MASLD is about 30%, death rates from extrahepatic cancers in MASLD far exceed those from hepatocellular carcinoma. Of note, the increased extrahepatic cancer risk in MASLD is not dependent on the liver fibrosis stage. Thus. the burden of MAFLD is going to be huge in the near future in terms of access to care, and social costs. 

## 3. Pathogenesis

The pathogenesis of MASLD (and specifically MASH) is complex and results from the interactions of genetic and environmental factors. This interplay leads to systemic metabolic dysfunction involving the liver, with deranged molecular pathways and cell-cell communication between hepatocytes, liver sinusoidal cells, stellate cells, Kupffer cells, and recruited immune cells [17]. Variable combinations of these factors may generate highly different clinical phenotypes among individuals with MASLD. For this reason, finding a single effective treatment has become a major challenge, while the need to adequately characterize subjects with MASLD is also justified for the goal of developing personalized management of the disease.

### 3.1. Genetic Aspects

Genetic predisposition accounts for the alteration of molecular pathways in liver cells, such as intrahepatic lipolysis, export of triglycerides, mitochondrial oxidation, and glycolysis [41,42,43,44,45,46]. The variant in the “patatin-like phospholipase domain-containing 3” (*PNPLA3*) gene on chromosome 22 is associated with modifications of retinol metabolism and variable manifestations of MASLD [46,47,48]. Single nucleotide polymorphisms (SNPs) in genes involved in insulin resistance have also been described in individuals with MASLD, including the genes of “ectoenzyme nucleotide pyrophosphate phosphodiesterase 1” (*ENPP1* or *PC1*) and “insulin receptor substrate-1” (*IRS1*) [49]. The variant in the “transmembrane 6 superfamily member 2” (*TM6SF2*) gene on chromosome 19 appears to be associated with an impaired lipid transporter and fatty liver disease [50]. SNPs in genes involved in oxidative stress and increases the risk of fibrosis, i.e., “membrane-bound O-acyl-transferase domain-containing 7” (*MBOAT7-TMC4*) genes, have also been described in patients with MASLD [51]. Additional findings involve genetic variants in the genes of “glucokinase regulatory protein” (*GCKR*) [52], “solute carrier family 2-member 1” (*SLC2A1*) [53], and “17-beta hydroxysteroid dehydrogenase 13” (*HSD17B13*) [54].

### 3.2. Derangement of Lipid Homeostasis

Metabolic alterations develop at different levels and involve the abnormal expansion of visceral adiposity, an important site of lipid accumulation when subcutaneous adipose tissue capacity is surpassed. This condition is predisposing to increased efflux of free fatty acids (FFA) to the liver. Further steps include the development of insulin resistance with activation of pro-inflammatory cytokines and hormones, and qualitative and/or quantitative changes of gut microbiota, predisposing to “leaky gut” and impaired gut–liver axis [17,55,56,57,58]. The role of diet in increasing the influx of dietary FFA and carbohydrates is also outstanding. Under such systemic changes, the liver becomes the major target organ dysfunction due to modified insulin signaling, lipogenesis, and mitochondrial/microsomal dysfunction [58,59,60].

In MASLD, hepatocellular lipid accumulation, mainly in the form of triglycerides, is the consequence of the imbalance between lipid input and output. Free fatty acids (FFAs) represent the main substrate for triglyceride synthesis by esterification [61]. Upon excessive FFA accumulation in the liver, a cascade of harmful consequences includes lipo-toxicity [62,63,64], mitochondrial and endoplasmic reticulum dysfunction [65], activation of signaling pathways related to metabolism and inflammation [66], and receptor activation which will promote inflammation [67]. Not only FFAs but also de novo lipogenesis (DNL) intermediates, including diacylglycerol, are responsible for further disruption of metabolic homeostasis [68,69]. These steps result in excessive production of reactive oxygen species (ROS), which originate from impaired mitochondrial function [70,71]. At least initially, FFAs are esterified and transported via serum very low-density lipoprotein (VLDL) and/or oxidized with conversion to other substrates. This initial defense mechanism ultimately fails if FFAs excess influx overwhelms the mitochondria’s capacity to metabolize FFAs, leading to increased ROS production. This is the first step that accounts for the potential evolution from simple steatosis to MASH [71,72,73].

**Figure 2:**
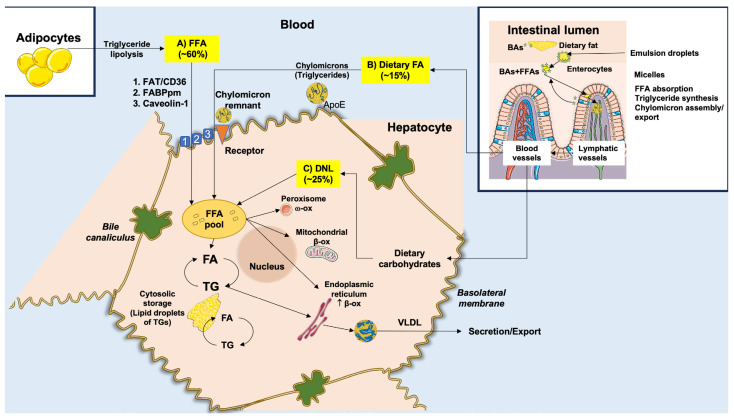
Origin and metabolism of free fatty acids (FFAs) in the liver. FFAs are supplied to hepatocytes from three major sources. (A) About 60% of the total FFA pool derives from the uptake of circulating FFAs that originate from the lipolysis of triglycerides (TGs) in the adipose tissue. FFAs enter hepatocytes across specific transporters, such as (1) fatty acid translocase/cluster of designation 36 (FAT/CD36) transporter, (2) fatty acid binding protein (FABPpm), and (3) caveolin-1. (B) About 15% of the total FFA pool derives from dietary FFAs. In the intestinal lumen, within enterocytes, FFAs are incorporated into TGs of chylomicrons, following ingestion of fat, with the help of conjugated bile acid (BAs) micelles. Chylomicron remnants are taken up by specific receptors in the hepatocyte with a high affinity for ApoE. (C) About 25% of the total FFA pool originates within the hepatocytes from de novo lipogenesis (DNL), utilizing dietary carbohydrates. The hepatocellular FFA pool can undergo peroxisome ω-oxidation, mitochondrial β-oxidation, endoplasmic reticulum β-oxidation, or re-esterification with glycerol to form TGs. TGs can be stored in lipid droplets in small amounts (<5%) or exported into the circulation as very-low-density lipoproteins (VLDL) which are assembled in the endoplasmic reticulum. Right inlet: stars represent BAs. Adapted from Di Ciaula et al. [41].

FFAs originate from three main sources (Figure 2): (a) spontaneous lipolysis of adipose tissue contributes to about 60% of the total influx of FFAs to the liver; (b) DNL contributes to about 25%; and (c) excessive dietary FFAs will contribute to about 15%. The pathway of lipolysis starts with the production of cyclic adenosine monophosphate (cAMP). Protein kinase A (PKA) is then activated to phosphorylate specific lipases, namely phospho-hormone-sensitive lipase (p-HSL) and phospho-perilipin 1 (p-PLIN1). Insulin inhibits this pathway [74].

**Figure 3:**
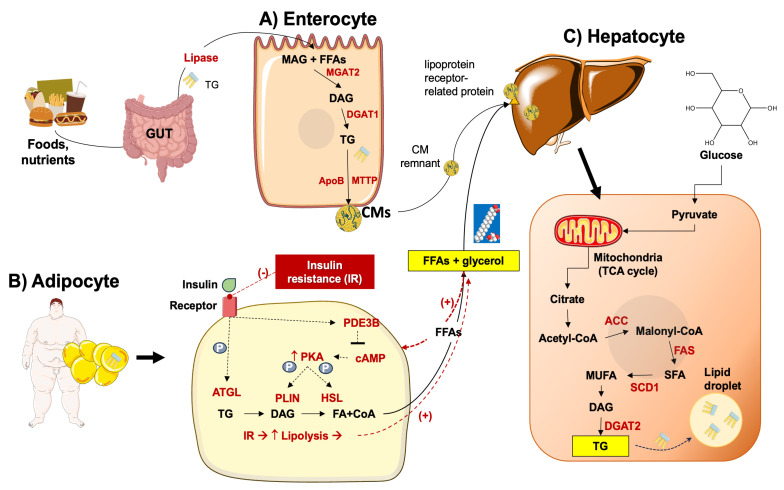
Lipid metabolism in MASLD. (A) At the gut level, lipase facilitates the breakdown of triacylglycerol (TG) into monoacylglycerol (MAG) and free fatty acids (FFAs) which in the enterocytes are re-synthesized into TG through enzymatic processes mediated by mannoside acetylglucosaminyltransferase (MGAT2) and diglyceride acyltransferase (DGAT1). TGs are transferred to chylomicrons (CMs) via the microsomal triglyceride transfer protein (MTTP), and transported via the lymphatic vessels to the liver, where remnants of CMs are absorbed post-lipolysis [80,81]. (B) In the adipocyte, insulin plays a pivotal role in lipid storage by suppressing lipolysis through the inhibition of adipose triglyceride lipase (ATGL), phosphodiesterase 3B (PDE3B), and hormone-sensitive lipase (HSL) regulated by protein kinase A (PKA) and perilipins (PLINs). However, in insulin-resistant states such as obesity or type 2 diabetes mellitus (T2DM), reduced insulin sensitivity fosters heightened lipolysis, resulting in an increased flux of FFAs to the liver. (C) In the liver, various key enzymes govern the de novo lipogenesis of saturated fatty acids (SFA), monosaturated fatty acids (MUFA), diacylglycerol (DAG), TG, and include including acetyl-CoA carboxylase (ACC), fatty acid synthase (FAS), stearoyl-CoA desaturase (SCD1), and DGAT2. Another important pathway includes the transformation of glucose to pyruvate, which then enters the mitochondrial tricarboxylic acid cycle (TCA), with the production of citrate [82].

Following lipolysis, circulating FFAs are directed to the liver [75]. In MASLD, these mechanisms are upregulated in the adipose tissue and are independent of the presence of diabetes [76,77,78]. If obesity develops in the background of adipocyte hypertrophy and insulin resistance, lipolysis will also increase with excessive transport of FFAs to the liver (Figure 3) [79].

DNL utilizes glucose-derived acetyl-CoA subunits [83] condensed with a glycerol backbone [84]. Proteins involved in the transcriptional regulation of DNL are the sterol response element binding protein (SREBP1c) and the carbohydrate response element binding protein (ChREBP) [85,86]. This process develops along with the genetic upregulation and activation of FA synthase (FAS), acetyl-CoA carboxylase (ACC), and stearoyl-CoA desaturase 1 (SCD1). Acetyl-CoA is then transformed into malonyl-CoA via the catalytic activity of ACC [87]. Acyl carrier protein (ACP), a component of the FAS domain, transports malonyl-CoA to the prosthetic phosphopantetheine group of the acyl carrier protein (ACP) [88,89,90], and the elongating FA chain is transported to different catalytic centers at the active site cleft of FAS by its rotation [91,92,93]. This reaction contributes to the elongation of the 16- or 18-carbon FFA chain [94,95], the initial step of triacylglycerol (TG) synthesis. FFAs are incorporated into glycerol-3-phosphate via primary acylation. The result is lysophosphatidic acid (LPA) via glycerol-phosphate acyl transferase (GPAT) [84]. The desaturated acylglycerol-phosphate acyl transferase catalyzes LPA to phosphatidic acid (PA). PA is dephosphorylated by phosphatidic acid phosphorylase (PAP) and diacylglycerol (DG) is produced [96]. The catalytic activity of diacylglycerol acyl-transferase (DGAT) results in DG acylated to TG [80]. The process of DNL increases FFA synthesis and inhibits β-oxidation by the intermediate product malonyl coenzyme [97].

In the gut, the pancreatic lipase transforms the dietary triacylglycerol into FFAs and monoacylglycerol. In the enterocytes, triacylglycerol is resynthesized by monoacylglycerol acyltransferase 2 (MGAT2), and then by DGAT. Triacylglycerol is incorporated into chylomicrons which are secreted into lymphatic vessels. FFAs, after catalysis by lipases, are stored in adipose tissue or utilized by muscle tissue as an energy source. After lipase catalyzation, chylomicron (CM) remnants are absorbed by the liver [98], whereby FFAs form triglycerides and become incorporated into VLDL particles to be exported into the bloodstream (Figure 3) [80,99]. If the absorption of CM remnants increases, the accumulation of lipids in the liver also increases [100,101].

There is evidence that nuclear receptors can be activated or become dysfunctional in MASLD, as found for the bile acid (BA) receptor farnesoid X receptor (FXR) [102,103], the liver X receptor (LXR) [104], the pregnane X receptor (PXR) [105], and the vitamin D receptor (VDR) [106]. Deranged nuclear receptors can contribute to the onset of hepatic inflammation and activation of abnormal inflammatory pathways. In addition, the activation of hepatic stellate cells triggers a fibrogenic response with the production of an extracellular matrix [107].

Dietary factors involving excess consumption of carbohydrates and saturated fat lead to metabolism imbalance at the level of the liver and skeletal muscle. In particular, lipid deposition increases in skeletal muscle, and this step, together with the onset of insulin resistance, leads to increased intramyocellular lipid content and to parallel inhibition of storage of ingested glucose as muscle glycogen [108]. As a consequence, glucose is rerouted to the liver on the background of ongoing insulin resistance, which will further promote DNL. Activated transcription factors are SREBP1c [109] (with increased VLDL production and hypertriglyceridemia), the carbohydrate-responsive element–binding protein (ChREBP) [110], the LXR [111], and the peroxisome proliferator-activated receptor gamma coactivator 1-beta (PPARg coactivator 1-b) [112]. Additional factors involved in the pathogenesis of MASLD include increased levels of endogenous lipoprotein lipase (Lpl) inhibitors, with a decreased clearance ability of circulating triglyceride-enriched lipoproteins by Lpl [113]. Lpl inhibitors include apolipoprotein C3 (ApoC3) [114], angiopoietin-like proteins 3/8 (ANGPTL3/8) complex, and ANGPTL4, among others [115]. These molecular mechanisms collectively promote the hepatocellular uptake of triglycerides.

### 3.3. Derangement of Carbohydrate Homeostasis

**Figure 4:**
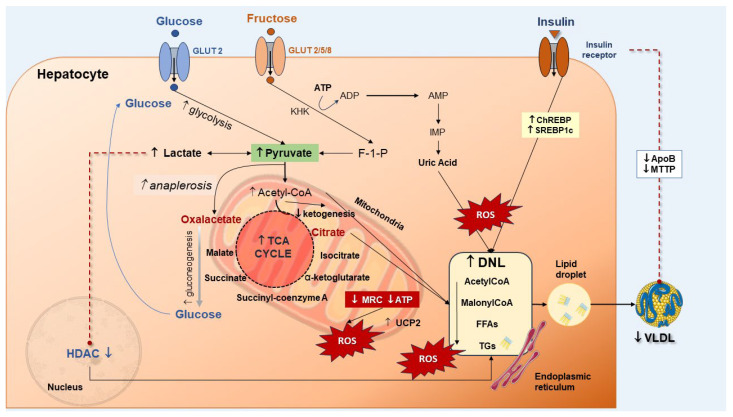
The interplay between glucose, fructose, insulin, and de novo lipogenesis (DNL) in MASLD. Increased glucose transport into the hepatocyte increases the glycolysis and pyruvate synthesis which contributes to the tricarboxylic acid (TCA) cycle. Increased pyruvate can be converted either to lactate or oxaloacetate via anaplerosis [127]. Conversion of pyruvate to lactate inhibits the histone deacetylase (HDAC) activity, thereby stimulating DNL. Production of oxalacetate is associated with increased gluconeogenesis, glucose production, and DNL. Both oxaloacetate and lactate are enhanced in MASLD [41,128]. Fructose enters the hepatocyte and is rapidly phosphorylated to fructose-1-phosphate (F-1-P) by the ketohexokinase (KHK). Adenosine triphosphate (ATP) hydrolysis to adenosine diphosphate (ADP), to adenosine monophosphate (AMP) and inosine monophosphate (IMP) provides increased uric acid levels which further contributes to DNL. Increased insulin upregulates the liver carbohydrate-responsive element-binding protein (ChREBP) and the sterol regulatory element-binding protein 1 (SREBP-1), which increases DNL with free fatty acids (FFAs) storage as triglycerides (TGs). Insulin also reduces very-low-density lipoprotein (VLDL) production through downregulation of the synthesis of the microsomal triglyceride transfer protein (MTTP) and apolipoprotein B (ApoB) [41]. Production of reactive oxygen species (ROS) which promote inflammation and hepatocellular injury can depend on increased glycolysis and FFA oxidation with acetyl-CoA abundance and enhanced activity of the TCA cycle. At the same time, ketogenesis is reduced. Moreover, the activity of the mitochondrial respiratory chain (MRC) is reduced, increasing the ROS generation. Uncoupling protein (UCP2) expression increases in MASLD. This step is associated with impaired efficiency of ATP synthesis and decreased ATP content [127].

Glucose metabolism is involved in the onset and progression of MASLD/MASH [116,117]. MASLD patients have increased levels of enzymes governing glycolysis which lead to enhanced glycolytic capacity such as hexokinase 2 (HK2) and pyruvate kinase isozyme type M2 (PKM2). One consequence of MASLD is the abnormal accumulation of triglycerides in hepatocytes [118,119]. Upon an increase in glucose transport to the liver, glycolysis will increase. Pyruvate is converted to oxaloacetate and more substrates become available for DNL. Alternatively, pyruvate can be converted to lactate, which decreases the activity of histone deacetylase (HDAC), a step stimulating the DNL pathway [118,120] (Figure 4). Increased levels of lactic acid stimulate the uptake of FFAs by hepatocytes and promote the expression of lipogenic genes [120]. A further step leads to oxidative stress and DNA damage in the stage of steatohepatitis with mitochondrial dysfunction and a deranged TCA cycle [41,121,122]. Insulin resistance, either hepatic or systemic, has negative reflections on MASLD. Hepatic insulin resistance can be triggered by short-term consumption of high-fat diets, and this condition develops in the absence of peripheral insulin resistance [123]. A consequence of insulin resistance is the disinhibition of gluconeogenesis [79], resulting in increased production of glucose [124] and increased DNL. The effects of insulin in promoting DNL involve the activation of the liver X receptor (LXR), with upregulation of *Chrebp1* and *Srebp1* genes [104]. Insulin also inhibits microsomal triglyceride transport protein (MTTP) and promotes apolipoprotein B (ApoB) degradation, both involved in the regulation of VLDL production. With insulin resistance, the uptake of FFAs increases in the liver, along with decreased phosphorylation of forkhead box transcription factor 1 (FoxO1), with the increase in MTTP [125] and the degradation of ApoB (Figure 4) [126].

Fructose has profound effects on metabolic homeostasis [129]. It is another player in the pathways involved in MASLD and can worsen hepatic steatosis [130]. At variance with glucose, fructose in the liver is catalyzed by phosphofructokinase, a step that leads to increased substrates for the DNL pathway [130]. There will be a continuous decrease in ATP and phosphate [131,132,133,134] leading to an increase in uric acid, ATP deficiency [135], inhibition of protein synthesis, and increased oxidative stress [134,136]. Fructose is able to stimulate the DNL pathway while inhibiting β-oxidation through ChREBP and SREBP1c, and less FFA consumption [136,137]. These steps worsen steatosis (Figure 4). Additional effects of fructose include the potential for induction of gut dysbiosis, further production of SCFAs, and increase in intestinal permeability, a condition which increases the endotoxin flow to the liver [138,139,140].

### 3.4. Deranged Bile Acid Homeostasis

The gut microbiota has a role in maintaining systemic homeostasis, and this function is partly dependent on the metabolism of bile acid (BA)-mediated signal transduction together with specific receptors [141]. Disrupted BA metabolism and function contribute to the damage observed in chronic liver diseases [103], which also includes the sequence of steatosis to steatohepatitis, independently of obesity and diabetes [142].

**Figure 5:**
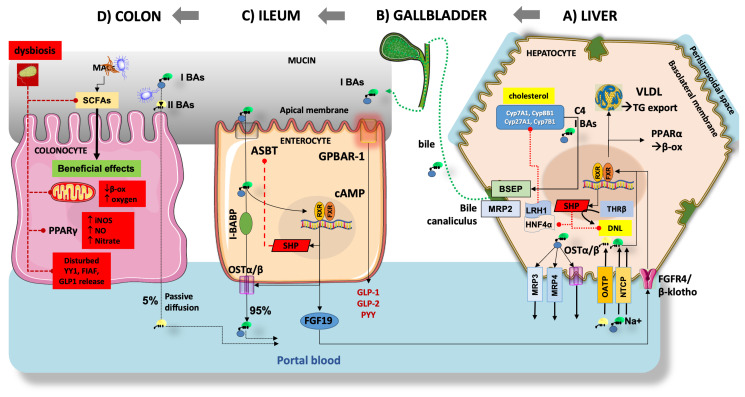
The interaction between bile acid (BAs) and gut microbiota is shown in the liver, the gallbladder, the terminal ileum, and the colon. Derangement of pathways at several levels can play a role in MASLD (see text for details). (**A**) Starting from cholesterol in the hepatocyte, the classical pathways use the oxysterol 7α-hydroxylase (CYP7A1), and CYP8B1, resulting in 7α-OH-4-cholesten-3-one (C4) and then “primary” BAs (I BAs) cholic acid (CA) and chenodeoxycholic acid (CDCA). The alternative pathway relies on CYP27A1 and CYP7B1, resulting in small amounts of CDCA [169,170]. Primary BAs are promptly conjugated (symbol O) with taurine and glycine, to increase solubility in bile [102]. The transport of BAs from the hepatocyte includes several pathways. Approximately 5% of BAs are transported to the systemic circulation via the multidrug resistance-associated protein 3 (MRP3), MRP4, and the organic solute transporter (OSTα/β). Basolateral import of BAs is mediated by sodium taurocholate co-transporting polypeptide (NTCP) (Na+-dependent) and organic-anion-transporting polypeptide (OATP) isoforms (Na+-independent). Intracellular BAs contribute to the negative feedback regulation of BA synthesis via the activation of farnesoid X receptor (FXR)-retinoid X receptor (RXR)-dependent pathways. These pathways increase the small heterodimer partner (SHP) expression and inhibit the hepatocyte nuclear factor 4α (HNF4 α) and nuclear receptor liver receptor homolog-1 (LRH1) which, in turn, leads to decreased activity of CYP7A1 and CYP8B1 [171]. Activation of the FXR-SHP pathway also inhibits de novo lipogenesis (DNL), promotes peroxisome proliferator-activated receptor α (PPARα) β-oxidation, and stimulates very-low-density lipoprotein (VLDL) production and TG export [144,145]. The nuclear thyroid hormone receptor β (THRβ) also contributes to DNL and works in concert with the above-mentioned nuclear receptor pathways. Conjugated BAs are secreted in bile canaliculus by the bile salt export pump (BSEP) and multidrug resistance-associated protein 2 (MRP2), and aggregate as micelles and vesicles with secreted cholesterol and phospholipids. (**B**) Bile enters the gallbladder to be temporarily stored and concentrated during fasting. Upon consumption of a fat-enriched meal, the cholecystokinin release prompts gallbladder contraction and secretion of bile/BAs into the duodenum. (**C**) In the terminal ileum, approximately 95% of BAs undergo reabsorption by enterocytes through the apical sodium-dependent bile salt transporter (ASBT), transported via the ileal bile acid-binding protein (I-BABP), and subsequently excreted into the portal vein via OSTα/β [172]. In humans, the BA-induced activation of ileal FXR has several consequences, including the activation of SHP with inhibition of ASBT, the RXR-mediated activation of OSTα/β and the fibroblast growth factor 19 (FGF19) production and secretion into the portal blood. Upon reaching the liver, FGF19 binds the liver FGFR4/β-klotho receptor with effects on FXR, with the above-mentioned effects on BA synthesis and DNL [173]. In the ileum, the activation of the membrane BAs receptor G-protein coupled BA receptor-1 (GPBAR-1) increases the cyclic adenosine monophosphate (cAMP) and increases the secretion of glucagon-like peptide-1 (GLP-1), GLP-2 and peptide YY (PYY) leading to a number of systemic metabolic effects. (**D**) In the colon, small amounts of primary BAs undergo bacterial biotransformation to unconjugated secondary BAs (II BAs) deoxycholic acid (DCA), and lithocholic acid (LCA) which are passively transported back to the liver. Under healthy conditions, undigestible dietary fibers represent the microbiota accessible carbohydrates (MACs). These are fermented by the local microbiota and produce short-chain fatty acids (SCFAs), mainly butyrate, acetate, and propionate. SCFAs are actively transported in the colonocyte to produce local beneficial effects, including anaerobic conditions maintenance through β-oxidation, decreased nitrate production, and balanced metabolic homeostasis in conjunction with peroxisome proliferator-activated receptor gamma (PPARγ). SCFAs also contribute to metabolic stability through the secretion of GLP-1, fasting-induced adipose factor (FIAF), and Yin-Yang 1 (YY1) [174,175]. These mechanisms are highly impaired at the onset and progression of MASLD and gut dysbiosis (red pathways).

Figure 5 depicts the main pathways regulating BA homeostasis in the entero-hepatic circulation. Inhibiting the ileal/colonic reabsorption of BAs partly interrupts the enterohepatic circulation of BAs and enhances their fecal excretion. This, in turn, prompts the conversion of more cholesterol to BAs, thereby reducing the risk of obesity [143]. Particular relevance has the hormonal function of BAs as ligands for FXR and the membrane-associated G protein-coupled bile acid receptor 1 (GPBAR-1, previously known as Takeda G protein-coupled receptor 5, TGR5) during their absorption in the terminal ileum [102]. In the liver, FXR deactivates the lipogenesis pathway by inhibiting SREBP1c. Furthermore, FXR induces β-oxidation by activating peroxisome proliferator-activated receptor-α (PPARα) and facilitates the clearance of VLDL in plasma, ultimately improving metabolic dysfunction in MASLD [144,145,146]. Furthermore, hepatic FXR promotes the oxidation of FFAs and ketogenesis, a process that relies on fibroblast growth factor 21 (FGF21) [147,148]. In parallel with these events, the activation of intestinal FXR induces intestinal epithelial cells to release FGF15/19 into the liver, effectively diminishing hepatic steatosis and enhancing insulin resistance [149,150,151,152]. The impact of FXR on MASLD remains a topic of debate, due to its widespread distribution in various tissues. Recent findings indicate that global knockout of FXR resulted in improved insulin sensitivity in *ob/ob* and high-fat diet (HFD) mice. This improvement might be attributed to the notion that prolonged activation of FXR diminishes energy expenditure and exacerbates HFD-induced glucose intolerance (Figure 5) [153,154,155]. In mice with liver-specific FXR knockout, however, this effect was not observed, suggesting a significant contribution by intestinal FXR [156]. Concurrently, it has been shown that elevations in the level of T-β MCA, an intestinal FXR antagonist, improve MASLD by enhancing the synthesis of BAs [157,158,159]. Additionally, the activation of intestinal FXR leads to a reduction in GLP-1 secretion [160]. Therefore, the role of intestinal FXR in preserving metabolic homeostasis requires further validation (Figure 5). Another BA receptor, GPBAR-1, is predominantly expressed in the gallbladder, adipose tissue, intestine, and liver, and is activated primarily by secondary BAs [161]. Once GPBAR-1 is activated in muscles or brown adipose tissue, it stimulates energy consumption, and in the intestine, it increases the secretion of GLP-1 (Figure 5) [103,162,163]. Recent studies also found that GPBAR-1 has beneficial effects on MASLD-related hypothyroidism, regardless of the level of thyroid hormone [164]. Thyroid hormone β receptor (TRβ) regulates the synthesis of BAs by interfering with SHP [165,166] or CYP7A1 directly in the liver [167]. It has also been reported that activation of TRβ reduces systemic lipid content and increases lipid oxidation to improve hepatic lipid homeostasis [168].

### 3.5. Gut Dysbiosis

The gut microbiota is an important player in regulating metabolic homeostasis, and deranged bacterial populations or the onset of dysbiosis can be a predisposing condition for NAFLD [56,57,175]. Indeed, the microbiota profile can differ between obese and lean individuals [55]. The gut microbiota consists of trillion of bacteria that contribute to intestinal barrier protection, selective permeability and immune responses, and maintenance of metabolic balance within the host [176]. Such beneficial effects require the fiber-dependent transformation of gut carbohydrates to SCFAs [177]. Either qualitative and/or quantitative changes of microbiota in response to acute or chronic conditions can evolve as dysbiosis and promote a variety of metabolic disorders [55]. Over-nutrition stands out as a pivotal factor that can influence the composition of the gut microbiota [178]. Chronic low-grade inflammation is a recognized characteristic of MASLD. Inflammatory mediators, including endotoxin, originate from the gut microbiota [179], especially in the context of a high-fat diet, where endotoxin level is increased [180,181]. Recent evidence points to the important role of gut microbiota in MASLD [57,60]. Indeed, a high-fat diet leads to an elevation in certain bacteria, such as *Enterobacter cloacae B29, Escherichia coli py102*, and *Klebsiella pneumoniae A7*. These specific bacteria have been identified as contributors to NAFLD progression [182]. Furthermore, at the advanced stage, populations like *Proteus* and *Escherichia coli* can increase, whereas the abundance of *Firmicutes* was notably reduced [183]. *Ruminococcaceae* and *Veillonellaceae* have been associated with an increased risk of liver fibrosis [184]. Research has indicated that gut dysbiosis, particularly when dominated by *Enterobacteriaceae, Escherichia coli*, and *Shigella*, is linked to the progression of MASLD [185].

A recent study examined gut microbiota in subjects with morbid obesity undergoing bariatric surgery [186]. Subjects with histologically confirmed steatosis/MASH showed microbiota enrichment with *Enterobacteriaceae*, i.e., ethanol-producing bacteria, *Acidaminococcus* and *Megasphaera*, and depletion of *Eggerthellaceae* and *Ruminococcaceae*, i.e., SCFAs-producing bacteria. The microbiota patterns changed in subjects with hepatic steatosis, necroinflammatory activity, or fibrosis, mainly in terms of increased *Enterobacteriaceae* and decreased *Ruminococcaceae*. Specifically, *Escherichia coli* was associated with steatosis and necroinflammatory activity, and *Escherichia-Shigella* was associated with fibrosis and necroinflammatory activity [186]. 

Several studies have shown an association between metabolic dysfunction and reduced concentrations of bacteria responsible for producing SCFAs, specifically propionate and butyrate [187]. Butyrate has the potential to serve as a substrate, promoting β-oxidation to sustain an anaerobic environment crucial for the microbiota [188]. Butyrate suppresses the expression of nitric oxide synthase through the nuclear receptor peroxisome proliferator-activated receptor gamma (PPARγ). This leads to a reduction in nitric oxide (NO) which, in turn, hinders the growth of *Enterobacteriaceae* [189,190]. Butyrate can mitigate inflammatory conditions by activating immune cells, specifically regulatory T cells (Tregs) [191]. Furthermore, SCFAs play a beneficial role in preserving intestinal permeability and enhancing insulin secretion and sensitivity by promoting the increased secretion of glucagon-like peptide-1 (GLP-1) and peptide YY (PYY) (Figure 5) [174,192,193]. Gut dysbiosis may increase the severity of MASLD due to a decrease in SCFAs [194]. Particularly, diminished levels of SCFAs are associated with a decreased abundance of *Faecalibacterium prausnitzii* [195], *Akkermansia Muciniphila* [195], and *Dysosmobacter welbionis* [196]. Furthermore, disruption of the gut microbiota inhibits the ability of intestinal epithelial cells to release a lipoprotein lipase inhibitor, the fasting-induced adipose factor (FIAF), thereby leading to elevated levels of FFAs in the liver [175].

## 4. Therapeutic Management of MASLD

**Figure 6:**
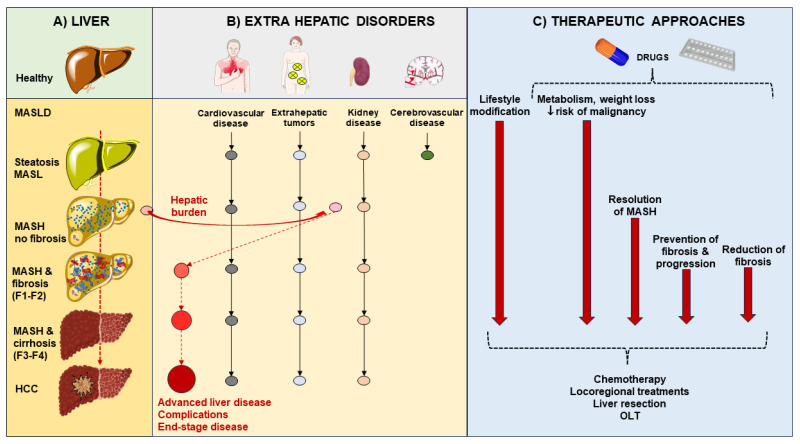
Potential progression of metabolic dysfunction-associated steatotic liver disease (MASLD) phenotypes in accord with extrahepatic disorders and therapeutic approaches. (**A**) Starting from the healthy liver, the hepatic burden of MASLD consists of simple steatosis (metabolic dysfunction-associated steatotic liver, MASL), necro-inflammatory status (metabolic dysfunction-associated steatohepatitis, MASH), fibrosis, cirrhosis and hepatocellular carcinoma (HCC). Fibrosis stages F1–F4 are reported. (**B**) The extrahepatic disorders are depicted, with the main concerns as cardiovascular disease, extrahepatic tumors, and kidney disease. As soon as MASH is demonstrated, the hepatic burden of the disease moves forward and becomes a main concern because of the potential progression to advanced liver disease, complications, and end-stage disease. (**C**) The mainstay of therapeutic approaches whenever possible consists of early prevention (lifestyle modification) of both hepatic and extrahepatic disorders. At a later stage, the use of precision medicine consists of personalized drugs targeting metabolism, body weight, and risk of malignancy. With MASH, further therapeutic approaches are aimed at the resolution of MASH, prevention of fibrosis and progression, or reduction of fibrosis. With cirrhosis and HCC, specific chemotherapy, locoregional treatments, liver resection and liver transplant (OLT) must be taken into account.

MASLD spectrum of disease is associated with several abnormalities which involve lifestyle, visceral adiposity, skeletal muscle, gut microbiota, and permeability, with dysregulated gut–liver axis [197]. Since the clinical course of MASLD is variable, therapeutic options consist of both general, metabolically-oriented, and liver-specific options (Figure 6). This latter option is especially indicated as the hepatic burden increases in individuals with disease activity testified by increased NAS, presence of NASH, and fibrosis who are at risk of fast progression to cirrhosis and liver-related events [35].

**Figure 7:**
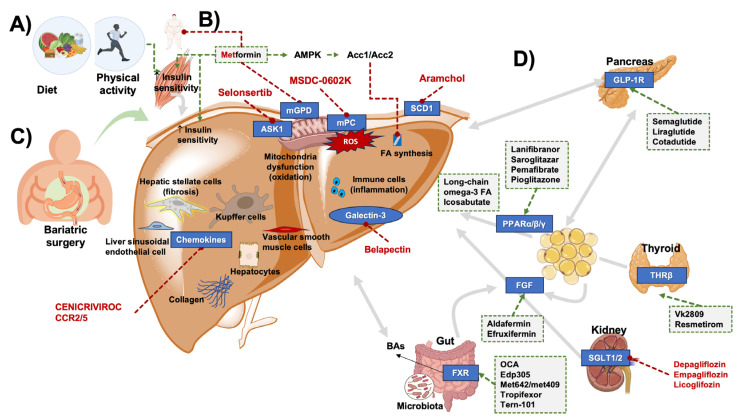
Potential therapeutic approaches in MASLD. Due to the complex interplay between pathogenesis, pathways, and organs involved, several options are being tested. (**A**) lifestyle including a healthy, balanced diet and regular physical activity improve insulin sensitivity and liver steatosis. (**B**) Whenever indicated, metformin can bring beneficial effects. (**C**) Bariatric surgery can play a role in the subgroup of severe obesity and increased cardiovascular risk. (**D**) With respect to drugs, effects can target the liver, several organs, and the microbiota, acting on specific pathways (see text for details). Green arrows indicate activation; red lines with dots indicate inhibition; grey arrows indicate interplay between organs. Abbreviations: Acc1/2, acetyl-CoA carboxylase 1, 2; AMPK, AMP-activated protein kinase; ASK1, apoptosis signal-regulating kinase-1; BAs, bile acids; FGF, fibroblast growth factor; FXR, farnesoid X receptor; GLP-1, glucagon-like peptide-1; mGPD, mitochondrial glycerophosphate dehydrogenase; mitochondrial pyruvate carrier (mPC); PPAR, peroxisome proliferator-activated receptor α/β/γ; ROS, reactive oxygen species; SGLT1/2, sodium-dependent glucose transporters 1,2; SCD1, stearoyl-CoA desaturase-1; THRβ, thyroid hormone receptor β.

Besides counterbalancing the caloric surplus and physical inactivity (possibly also through bariatric surgery or bariatric endoscopy), individuals with MASLD should be managed by improving the systemic metabolic homeostasis [198], dysregulation of glucose and lipid metabolism [82], and eventually correct the involvement of skeletal muscle (i.e., myosteatosis, sarcopenia, and release of myokines) [199,200], of intestinal dysbiosis [55,57], and leaky gut [60,176]. Systemic- and liver-specific therapy strategies can target liver fat deposition and insulin resistance, oxidative stress, endothelial cell injury, and inflammation. The overall spectrum of available treatments currently available is depicted in Figure 7. 

### 4.1. Lifestyle: Diet and Physical Exercise

Increased physical activity generates beneficial effects on metabolic disorders and associated conditions such as liver steatosis, and gallstone disease. These effects involve gut motility, the signaling role of BAs through their enterohepatic circulation, and a beneficial modulation of intestinal microbiota and inflammation [201]. Lifestyle remains the most evidenced approach in MASLD. However, it lacks long-term efficacy [202]. MASH might improve in about 58% of cases when body weight loss is >5% of initial body weight. The success rate is higher (about 90% of cases) if weight loss is >10% of initial body weight [203]. The dietary pattern must be tailored to a hypocaloric, low-fat, low-carbohydrate, or Mediterranean-type diet. Isocaloric diets with high protein content decrease hepatic steatosis and inflammation in T2DM patients [204]. 

A large systematic review and meta-analysis explored the effects of the Mediterranean diet and calorie restriction in patients with MASLD. Both dietary interventions improved hepatic steatosis and liver stiffness, with a dose-response relationship between the degree of calorie restriction and the beneficial effects in terms of liver function and weight loss [205]. A recent meta-analysis showed that intermittent fasting regimens lasting 2–3 months are generally able to reduce steatosis and ameliorate metabolic homeostasis, improving liver function in patients with MASLD. Evidence, however, is still derived from a limited number of studies, and the impact of diet needs to be further explored [206]. 

Exercise can reduce hepatic steatosis and improve liver stiffness [207], independently of dietary changes [208]. A moderate-vigorous exercise prevented fatty liver in 233,676 subjects enrolled in a five years follow-up [209]. The best results occurred in individuals exercising over 250 min a week [210]. The amelioration occurs in MASLD if the level of exercise is sufficient, regardless of whether aerobic exercise is performed [211]. Nevertheless, the effects of lifestyle interventions are rather slow, require high compliance in the medium- long-term [203,212], and can be counteracted by a number of factors, including stress and sedentary lifestyle, as reported in a study focusing on people during COVID-19 lockdown [213]. A recent meta-analysis in patients with biopsy-proven NAFLD found that exercise alone is not able to significantly improve NAFLD score or fibrosis, and does not lead to histopathological improvement [214]. In a recent paper exploring the outcome of obese subjects undergoing bariatric endoscopy [215], results obtained after lifestyle modification alone were poor. Despite decreased CAP values obtained by elastography (i.e., decreased extent of steatosis), results showed a modest weight loss (on average 4 Kg), unchanged BMI, and, of note, unmodified grade of liver fibrosis. Furthermore, in patients with fat over-storage, liver steatosis, and insulin resistance, diet, and physical activity had a minor role in the onset of subclinical liver dysfunction (i.e., decreased hepatic extraction efficiency, impaired liver microsomal function), as assessed by (^13^C)-methacetin breath test [59].

### 4.2. Bariatric Surgery

To date, there is debate regarding the adaptation of foregut bariatric surgery to MASLD treatment [15]. Bariatric surgery for correction of severe obesity-related comorbidities can include patients with MASLD but is not primarily performed for MASLD [216]. Some improvements in histological features, namely ballooning and lobular inflammation, are reported in about 75% of patients with steatohepatitis [217,218], despite a subgroup of patients being at risk of secondary steatohepatitis and liver fibrosis [219].

In a recent multicenter, open-label, randomized trial, 288 adult individuals with obesity with or without type 2 diabetes, and with histologically confirmed MASH were randomly assigned to lifestyle modification plus best medical care, Roux-en-Y gastric bypass, or sleeve gastrectomy [220]. As a primary endpoint, the authors searched for histological resolution of MASH without worsening of fibrosis at 1-year follow-up. The prevalence of participants meeting the primary endpoint was significantly higher in the Roux-en-Y gastric bypass group (54 [56%]) and sleeve gastrectomy group (55 [57%]) compared with lifestyle modification (15 [16%], intention-to-treat analysis). In the per-protocol analysis (236 [82%] participants who completed the trial), the primary endpoint was met in 54 (70%) of 77 participants in the Roux-en-Y gastric bypass group and 55 (70%) of 79 participants in the sleeve gastrectomy group, compared with 15 (19%) of 80 in the lifestyle modification group. Severe adverse events (6%) occurred in ten participants who had bariatric-metabolic surgery without re-operations. In this study, the authors show that bariatric-metabolic surgery is more effective than lifestyle interventions and optimized medical therapy in the treatment of MASH [220].

A recent meta-analysis involving 19 studies and a total of 911 patients evaluated the outcomes after positioning of intragastric balloon, a promising endoscopic bariatric therapy. Results showed beneficial effects on MASLD activity score, liver volume, and liver steatosis. These results were paralleled by decreased body weight, BMI, glycated hemoglobin, and extent of insulin-resistance [221].

### 4.3. Pharmacological Therapy

While the impact of lifestyle changes on MASLD is unquestionable, sustained results with weight management through dietary modification and physical exercise alone are notoriously difficult to achieve [222]. In the past decades, an increasing number of molecules targeting many different aspects of MASLD pathogenesis have been developed and tested [41,223,224]. Different biological targets have been identified according to the development and progression of the disease, with special attention to the histologically characterized disease pathophenotypes ranging from steatosis to inflammation to fibrosis. 

Among the molecules tested in the last 10 years for the treatment of MASLD/MASH, many have been abandoned before entering clinical trials or following phase 1 and 2 evaluation. Very few drugs have reached the level of phase 3 trial (Table 1). The most promising medications in the field include antidiabetic drugs, FXR agonists, PPAR agonists, and thyroid hormone receptor (THR) agonists (Figure 7). Most recently, the tide in the long quest for MASLD pharmacotherapy seems to be turning as resmetirom has become the first drug to be approved by the FDA in March 2024 for the treatment of MASH with significant (F2 or F3) fibrosis. While the approval of resmetirom as a liver-directed medication is indeed a culmination of many years of research, it is important to note that drug combinations likely represent the way forward in MASLD pharmacotherapy, promising increased efficacy rooted in the complex pathophysiology and reduced rates of adverse events [225].

#### 4.3.1. Antidiabetic Drugs

The use of antidiabetic drugs mainly oriented to counteract insulin-resistance in individuals with a steatotic liver has shown variable outcomes. In three randomized trials in diabetic and prediabetic patients with MASH, pioglitazone induced some histological amelioration by improving MASLD activity score (NAS) and a single inflammatory component of MASH. The effect on liver fibrosis was absent, and worsening of liver fibrosis was not observed [239,240,241]. 

The pharmacological effects of metformin on liver fat storage and metabolism are still scarcely explored [242] and the use of metformin to improve steatosis and steatohepatitis is controversial, due to contradictory findings [243,244]. A major effect of metformin is to inhibit gluconeogenesis, leading to decreased hepatic glucose output [245,246,247]. The mechanism likely involves the inhibition of mitochondrial glycerophosphate dehydrogenase (mGPD), a specific mitochondrial enzyme. The inhibition decreases the conversion of glycerophosphate to dihydroxyacetone phosphate and prevents glycerol from entering gluconeogenesis [248,249]. In addition, the inhibited mGPD increases the cytoplasmic NADH with decreased conversion of lactate to pyruvate, a step limiting lactate entering the hepatic gluconeogenesis. After a meal, metformin increases insulin-sensitivity and glucose utilization, especially in the skeletal muscle and liver [250]. Via its antilipolytic effect, metformin reduces the concentration of serum FFAs, a substrate for gluconeogenesis [250,251]. This effect involves the Peutz-Jeghers protein (LKB1)-dependent activation of the enzyme AMP-activated protein kinase (AMPK) in hepatocytes [252,253] which explains the AMPK-dependent inhibitory phosphorylation of acetyl-CoA carboxylases Acc1 and Acc2 and suppression of lipogenesis and FAs synthesis in liver and muscle [254,255]. Metformin likely decreases food intake and body weight [256,257].

In patients with new-onset T2DM, metformin generated, during 2 years, an improvement of the hepatic steatosis index but a worsening of liver fibrosis, determined by the FIB-4 index [258]. However, the ability to counteract insulin-resistance, but also to activate AMP-activated protein kinase (AMPK) [259] by inhibiting mitochondrial complex 1, reducing fatty acid synthesis, inducing mitochondrial fatty acid β-oxidation, reducing the production of ROS [260], and positively modulating gut microbiota in presence of metabolic disorders [55] can significantly improve the metabolic homeostasis, in particular in patients with T2DM and a steatotic liver [55,261]. As a result, although the use of metformin alone in treating subjects with liver steatosis is not convincingly supported, so far, a number of ongoing trials oriented to the management of the steatotic liver include the use of metformin together with other anti-diabetic drugs as SGLT-2 inhibitors, pioglitazone, liraglutide, gliclazide, sitagliptin [262]. In a recent Korean retrospective, nonrandomized interventional cohort study in patients with T2DM classified as NAFLD, SGLT2 inhibitors, thiazolidinediones, and DPP-4 inhibitors, each combined with metformin, have been associated with a clinical regression of NAFLD, when compared with the combination metformin- sulfonylureas [263].

Sodium-dependent glucose transporters-2 (SGLT-2) inhibitors function as potent sodium-dependent transporters of glucose after filtration from the kidney [264]. SGLT-2 inhibitors induce glucosuria, decrease glycemia and insulin levels (in particular in patients with T2DM), reduce hepatic de novo lipid synthesis [265], and contribute to weight loss and improved metabolism. This step is partly responsible for the indirect decrease in hepatic lipid accumulation since SGLT-2 is not expressed in the liver [264]. A number of studies have documented the beneficial effects of SGLT-2 inhibitors on the steatotic liver (including liver fibrosis), although the majority of observations are in patients with T2DM or included a limited number of subjects [266]. Among the oral SGLT2 inhibitors, dapagliflozin reduces hepatic lipid accumulation without significant effects on insulin sensitivity [239,267]. A meta-analysis of 7 trials explored by imaging patients with MASLD treated with dapagliflozin 10 mg, compared to placebo or control group. Treatment decreased both ALT and AST but not gamma-glutamyl transferase (GGT) serum levels. The degree of insulin resistance assessed by the homeostatic model assessment of insulin resistance (HOMA-IR) improved. Although levels of total cholesterol increased under dapagliflozin treatment, the safety profile between groups showed no significant difference [268]. The DEAN phase 3 trial (NCT03723252) aims to compare dapagliflozin vs. placebo in patients with histologically confirmed NASH. Primary endpoints are improvement in liver histology score at one year, improvement of MASH, changes in fibrosis score, and metabolic profile such as body weight, hemoglobin A1 (Hb1Ac), or insulin resistance. 

Other studies are currently evaluating the efficacy of other SGLT1/2 inhibitors in MASH, e.g., the ELIVATE study (NCT04065841) with licogliflozin alone or combined with the agonist of the BAs receptor FXR tropifexor on fibrosis and/or NAS score in patients with MASH and fibrosis stage 2 or 3. 

In patients with T2DM, empagliflozin reduced plasma levels of liver enzymes and decreased hepatic accumulation of lipids. This approach can represent a treatment in patients with T2DM and MASLD [269], and appears to reduce the risk of diabetic ketoacidosis and lower extremity amputation [270]. 

GLP-1 analogs and other incretins represent additional antidiabetic medications. Glucagon-like peptide 1 (GLP-1) is an endogenous gut hormone (incretin) that promotes insulin production and release, inhibits glucagon secretion indirectly, and, at the same time, reduces appetite. GLP-1 analogs are widely used in the treatment of T2DM. The receptor of GLP-1 (GLP-1R) is not significantly expressed in the liver, although it is widely distributed elsewhere [271]. The general metabolic improvement via GLP-1 includes insulin sensitivity, appetite suppression, and weight loss, and is likely responsible for the improvement in MASLD. In fact, a number of studies documented beneficial effects on the liver linked with the use of GLP-1 analogs, mainly in terms of regression of hepatic steatosis and improvement of NASH. Results, however, are less dramatic in terms of reduced hepatic fibrosis [266]. GLP-1 receptor analogs represent a promising tool primarily in patients with MASLD associated with T2DM and obesity. Seven different GLP-1 receptor agonists have been approved by the US Food and Drug Administration for the treatment of type 2 diabetes (i.e., exenatide, liraglutide, dulaglutide, albiglutide, lixisenatide, semaglutide, and tirzepatide). Among these, three (semaglutide, liraglutide, and tirzepatide) have been also approved for obesity and overweight. 

Exenatide, which is one of the first GLP-1R agonists, appears to directly increase hepatocyte uptake of glucose under oral glucose stimulation [228]. Exenatide stimulates the β-oxidation with downregulation of genes related to lipogenesis. This effect is beneficial to MASLD [272,273,274].

The LEAN phase 2 trial [227] reported, in patients with biopsy-confirmed MASH, histological resolution of MASH with liraglutide, another GLP-1 agonist, given at a dose of 1.8 mg daily compared to placebo. Fibrosis progression occurred in 9% and 36% of the liraglutide and placebo groups, respectively. A few side effects included gastrointestinal disorders and local reactions at the administration site. Liraglutide was effective in improving MASLD with 39% efficacy [275]. 

For the liver, semaglutide was first assessed in a phase 2 clinical trial lasting 72 weeks [226]. The target population was biopsy-proven MASH patients with liver fibrosis stages 1, 2, or 3, randomly assigned to semaglutide 0.1, 0.2, 0.4 mg or placebo. The study fixed as the primary outcome the resolution of MASH and no fibrosis worsening (only stage 2 or 3 fibrosis levels were assessed). The outcome was successful in 40%, 36%, 59%, and 17% of patients with 0.1, 0.2, 0.4 mg or placebo, respectively, meaning *p* < 0.001 for semaglutide 0.4 mg vs. placebo. The percentage of patients with improved fibrosis staging was not different from placebo (43% with 0.4 mg vs. 33% with placebo). Side effects, such as gastrointestinal and gallbladder disorders and an increase in amylase and lipase, were more common in the semaglutide groups. The ESSENCE phase 3 clinical trial (NCT04822181) is studying the effect of semaglutide on liver fibrosis in non-cirrhotic MASH patients. Results from phase 2 trials have waited for other GLP1R agonists such as cotadutide (NCT04019561), tirzepatide (NCT04166773), or efinopegdutide (NCT04944992).

#### 4.3.2. Statins

Within the wide background of metabolic abnormalities that are associated with MASLD, hyperlipidemia includes triglyceride-rich and cholesterol-rich lipoproteins in the serum. This is a condition pointing to the increased transport of lipids to the liver. Previous small clinical trials found that atorvastatin decreases ALT serum levels while improving hepatic steatosis [276], and that rosuvastatin reduces both ALT and AST serum levels with amelioration of liver fibrosis [277]. Recent clinical trials confirmed that statins can reduce the risk of hepatic steatosis and fibrosis [278] and improve MASLD/MASH expression [279]. More studies are awaited in this field.

#### 4.3.3. Peroxisome Proliferator-Activator Receptor (PPAR) Agonists

The family of PPAR α, γ, and δ receptors is located mainly in the liver, macrophages, and brown adipose tissue. In the liver, PPARs activate fatty acid oxidation, decrease the synthesis of TGs, and increase insulin sensitivity. In previous studies, the agonists of PPARs appear to improve NAFLD [280]. PPAR-γ influences the differentiation of adipocytes, regulates lipid and glucose metabolism, and inhibits inflammation [281]. Thiazolidinediones are potent activators of PPAR-γ and work as antidiabetic agents, in that they improve insulin sensitivity, reduce plasma FFAs, and hepatic lipid accumulation [282]. These molecules also improve fibrosis by inhibiting the activation of hepatic stellate cells [283]. Pioglitazone, a mild PPAR-γ activator, improves liver steatosis, and reduces liver enzymes but does not reduce fibrosis [284]. Its use is limited due to side effects such as weight gain and edema [285,286]. 

Pemafibrate is a PPARα agonist tested in MASH patients screened by MRI and ALT elevation [229]. Although the percentage change in liver fat content by MRI at week 24 was only −5.3% vs. −4.2% in controls, liver stiffness by MRI significantly decreased at week 48 and maintained at week 72 (treatment difference −6.2%).

Saroglitazar acts as a dual PPARα/γ agonist, first approved in India for the treatment of patients with T2DM and hypertriglyceridemia [287]. Saroglitazar was tested in MASLD patients diagnosed according to imaging (ultrasound, CT scan, or MRI), liver biopsy (MASH or simple steatosis), and biomarkers, i.e., ALT > 1.5 UNL (16 weeks Phase 3 trial EVIDENCES II) [235]. Saroglitazar 4 mg significantly reduced ALT levels (−45.8% vs. 3.4% treatment vs. placebo, respectively). MASH histology also significantly improved, showing decreased liver fat content (−19.7% vs. 4.1% with placebo). The safety and tolerability of saroglitazar were further assessed. Diarrhea and cough were the most frequent adverse effects, as reported in the phase 2 study EVIDENCES IV [288].

Elafibranor, a dual PPARα/δ agonist, reduced hepatic lipid accumulation, and improved inflammation and fibrosis [289]. Patients with obesity treated with elafibranor showed decreased liver enzymes and improved insulin sensitivity [290]. Elafibranor, however, failed to meet histological endpoints (i.e., NASH resolution, without worsening of fibrosis) and the secondary end-point (i.e., fibrosis improvement at least one stage) [291].

The pan-PPAR agonist lanifibranor can decrease the accumulation of hepatic lipids, liver enzyme levels, and biomarkers of plasma inflammation. In a 2b clinical trial, the drug improved liver fibrosis. Gastrointestinal side effects and weight gain were observed [292]. Volunteers are being recruited in a phase III trial. Lanifibranor is used in patients with non-cirrhotic, biopsy-confirmed highly active (stages 0–3) MASH (Phase 2b trial NATIVE). When assessing the histological resolution of MASH, the dose of 1200 mg was better than 800 mg or placebo (49% and 39%, respectively, vs. 22%). Similar results were evident for histological improvement of fibrosis (48% and 34%, respectively, vs. 29%), or both (35% and 25%, respectively, vs. 9%) [228]. Nausea, peripheral edema, anemia, diarrhea, and weight gain were the main side effects seen more frequently with lanifibranor than with placebo.

#### 4.3.4. FXR Agonists

FXR plays a key role in the pathways involved in BAs, glucose and lipid homeostasis [293], and regulation of inflammation [102,294,295,296,297]. A complex pathway involves the sequence governing BA synthesis. In particular, luminal BAs are re-absorbed into the ileal enterocyte and interact with the nuclear FXR with upregulation of fibroblast growth factor (FGF)-19 expression. In the process of enterohepatic circulation of BAs, FGF-19 binds the specific hepatic FGFR4/β-Klotho receptor and inhibits the expression of CYP7A1, the rate-limiting enzyme of BA synthesis [28,102]. This multilevel pathway is responsible for a protective effect against the toxic accumulation of BAs in the liver and in bile canaliculi. In addition, liver FXR activation ameliorates glucose tolerance due to reduced hepatic gluconeogenesis and increased synthesis of glycogen [298]. FXR activation reduces hepatic fat accumulation via SHP expression and CYP7A1 activity [299]. 

The semi-synthetic BA obeticholic acid (OCA), the analog of the primary BA chenodeoxycholic acid, has been tested due to a potent agonistic effect on FRX and possible beneficial metabolic effects on hepatic lipid and glucose metabolism. However, in a large phase 3 randomized, placebo-controlled trial designed to test the long-term effects of OCA on MASH and fibrosis in patients with stage 1–3 fibrosis, OCA treatment did not meet the endpoint related to MASH resolution. A dose-dependent pruritus was reported requiring treatment discontinuation in <10% of the cases and about 50% of patients in the OCA group developed hypercholesterolemia requiring newly prescribed statins [300].

Other synthetic FXR agonists are being tested and might produce fewer adverse events than OCA. MET642 is included in a phase 2 clinical trial enrolling NASH patients (NCT0477396). MET409 alone, in a phase 1b trial, reduced liver fat content, and currently is associated with empagliflozin in a phase 2b trial. The non-BA tropifexor is another highly potent FXR agonist tested in the FLIGHT FXR phase 2 clinical trial (NCT02855164) in MASH patients with stage 1–3 fibrosis. In an initial analysis, tropifexor safely reduced hepatic fat, liver transaminases, and body weight, as compared to placebo [301].

The nonsteroidal FXR agonist cilofexor 30 mg decreased liver steatosis and reduced the content of primary BAs without significant changes in liver fibrosis in a phase 2 trial. With 100 mg, however, moderate to severe pruritus occurred [233]. Acting at different therapeutic levels might be another therapeutic option, since in a phase 2 trial testing cilofexor combined with the acetyl-CoA carboxylase (ACC) inhibitor firsocostat and the antidiabetic GLP-1 RAs semaglutide improved liver steatosis and liver biochemistry [302].

The potential therapeutic role of ACC inhibitors in MASLD must be considered. Firsocostat reduces lipid accumulation while improving liver fibrosis. The effect on de novo lipogenesis is involved and a study achieved 12 weeks of intervention. The risk of hypertriglyceridemia is increased [303]. PF-05221304 is a potent and reversible dual ACC1/2 inhibitor. In a 16-week phase 2 clinical trial, a dose of 10 mg daily decreased lipid accumulation in the liver by up to 65%. However, serum TGs increased in 8% of subjects [304].

Other FXR agonists are being tested as well. EDP305, in a phase 2 randomized, double-blind, placebo-controlled, dose-ranging trial (ARGON-1), reduced liver fat content and decreased ALT in non-cirrhotic biopsy-proven MASH patients treated during a 12-week period [305]. Mild pruritus and changes in lipid parameters occurred. EDP305 is being tested in NASH patients with stage 2–3 fibrosis in the phase 2b study ARGON-2 (NCT04378010). TERN-101 is another nonsteroidal FXR agonist tested in the LIFT phase 2 trial. This treatment decreased ALT and GGT levels in patients with stage 1–3 liver fibrosis after a 12-week course. TERN-101 beneficially affected inflammation and fibrosis measured by a non-invasive composite marker including MRI (cT1) and MRI-PDFF [306].

Fibroblast growth factor (FGF) analogs can also play a role in the therapy of MASLD. The FGF superfamily includes FGF19 and FGF21, which have beneficial effects on glucose and lipid metabolism. In animal models, the administration of FGF19 and FGF21 improves insulin sensitivity, lipid levels, and liver steatosis while ameliorating body weight and fat mass. A potential explanation is the inhibition of SREBP1 and the reduced expression of genes involved in TG synthesis [307]. 

Aldafermin (NGM282) is a 190-amino-acid peptide and an engineered analog of recombinant human FGF19 with a 95.4% homology. Aldafermin can inhibit BAs synthesis and regulate metabolic homeostasis. In a 24-week phase 2b trial, aldafermin decreased hepatic lipid accumulation by 7.7% but liver fibrosis did not improve among patients with MASH-related stage 2 or 3 fibrosis [308]. Aldafermin was well tolerated in another phase 2b trial, without a significant dose-dependent response in fibrosis [309].

FGF21 secretion is dependent on starvation, nutritional stress, a high-fat diet, or a nutritional restriction diet [280,281]. In addition, FGF21 can modulate obesity and hepatic metabolic homeostasis via increased energy consumption and insulin sensitivity [282]. The ultimate mechanism accounting for the hepatoprotective effect of FGF21 is still unknown [283]. In a phase II trial, the FGF21 analog PEGylated pegbelfermin (PGBF), given subcutaneously for 16 weeks, decreased hepatic lipid accumulation in NAFLD patients. However, 16% of patients developed adverse effects, such as nausea [284]. The trial testing the effect of PGBF on fibrosis in MASLD needs to report data [286]. 

B1344 is a long-acting PEGylated FGF21 analog that significantly reduced hepatic steatosis, inflammation, and fibrosis in cynomolgus monkeys with MASLD undergoing liver biopsies [285].

Another FGF21 analog, efruxifermin, in a phase 2 trial reduced liver fat content, improved liver function tests, fibrosis and inflammation markers, and NAS score in patients with liver fibrosis stage 1–3. The fibrosis stage improved by at least 1 point in about 50% of the patients and resolution of MASH was seen in about 30%. The safety profile was favorable [232].

#### 4.3.5. Thyroid Hormone Receptor Beta (TR-β) Agonists

TR-β is also known as nuclear receptor subfamily 1, group A, member 2 (NR1A2), a nuclear receptor protein encoded by the *THRB* gene in humans. The protein is a sensor for triiodothyronine, improves hepatic regulation of lipid metabolism, plays a role in insulin sensitivity, promotes liver regeneration, and reduces hepatocyte apoptosis in the liver [223]. Pathways involved in the liver include the inhibition of the small heterodimer partner (SHP) and DNL, the activation of PPARα and β-oxidation, and the increased export of VLDL. TR-β also promotes liver regeneration and reduces hepatocyte apoptosis [310].

Selective THR-β agonists improve the conversion of T4 to T3 and likely enhance mitochondrial function, besides the anticipated beneficial metabolic effects [310,311,312]. The THR-β agonist resmetirom (MGL-3196) targets the liver and was used in a 36-week phase 2 trial in patients with MASH and fibrosis. Liver fat content decreased, and lipid metabolism parameters and liver function tests improved. In addition, lipid profile and fibrosis markers improved without affecting body weight [313]. In a phase 3 trial patients with a non-invasive diagnosis of MASLD received 100 mg/daily of resmetirom for 52 weeks. About 50% of treated patients and 8% of placebo-treated patients showed improved liver fat content while liver fibrosis improved in about 20% of treated patients vs. 10% of placebo-treated patients, as confirmed by a non-invasive test. With resmetirom, liver enzymes, lipid metabolism parameters, and inflammatory biomarkers also improve in the absence of major safety concerns. Diarrhea and nausea are the main adverse events [314]. In a phase 3 study in MASH patients with fibrosis (MAESTRO-NASH, NCT03900429), resmetirom use was associated with MASH resolution and improvement in liver fibrosis by at least one stage. In particular, 966 patients with biopsy-confirmed MASH and a fibrosis stage of F1B, F2, or F3 were randomly assigned in a 1:1:1 ratio to receive once-daily resmetirom (322 in the 80-mg resmetirom group, 323 in the 100-mg resmetirom group, and 321 in the placebo group) during 52 weeks. MASH resolution with no worsening of fibrosis was significantly achieved in 25.9% of the patients in the 80-mg resmetirom group and 29.9% of those in the 100-mg resmetirom group, as compared with 9.7% of those in the placebo group. Fibrosis improved by at least one stage with no worsening of the NAFLD activity score occurring in 24.2% of the patients in the 80-mg resmetirom group and 25.9% of those in the 100-mg resmetirom group, as compared with 14.2% of those in the placebo group. At week 24 LDL cholesterol levels decreased by −13.6% and −16.3% in the 80-mg and 100-mg resmetirom group, respectively, vs. 0.1% in the placebo group. Diarrhea and nausea were more frequent with resmetirom than with placebo. The incidence of serious adverse events was no more than 13%, and comparable across the three groups [310,315]. Based on these results, resmetirom received accelerated approval by the Food and Drug Administration on March 14, 2024, becoming the first-ever drug approved for the treatment of MASH with stage F2 or F3 fibrosis, 

VK2809, a liver-directed THR-β agonist, improved liver fat content in NAFLD patients treated with two different doses in a phase 2 trial [316]. Patients are being recruited in a phase 3 RCT.

#### 4.3.6. Anti-Fibrotic and Anti-Inflammatory Agents

Several additional compounds are tested for their anti-fibrotic and anti-inflammatory effects. Examples are GB1211 targeting galectin 3, DFV890 targeting NLPR-3, or nimacimab targeting CB1 tested in phase 1 or trialstipelukast, a leucotrien, or nitazoxanide, an antiparasitic agent in phase 2 trials. Results about safety and efficacy are therefore awaited.

Other drugs are being tested in more advanced trials, such as cenicriviroc (CVC) a small antagonist molecule administered orally, able to block chemokine 2 and 5 receptors, both involved in liver inflammation and fibrosis. CVC in the CENTAUR phase 2b trial, failed to achieve any histological improvement in NASH but CVC ameliorated liver fibrosis without worsening NASH [232]. In the AURORA phase 3 trial (NCT03028740) the interim analysis did not confirm the efficacy of CVC and the trial was prematurely interrupted. 

The TANDEM phase 2b trial (NCT03517540) reported that in patients with biopsy-proven NASH with fibrosis, combined treatment with CVC plus tropifexor was safe, decreased body weight and ALT. Of note, when considering the histological endpoints, the combination of CVC plus tropifexor was not superior to either drug in monotherapy [317].

Galectin-3 is a B-galactoside-binding lectin involved in inflammatory response and fibrosis. The complex carbohydrate belapectin targets Galectin-3 [318] but in the NASH-CX phase 2b clinical trial (NCT02462967) did not have a significant effect on inflammation and fibrosis compared to placebo. Belapectin, however, decreased the hepatic venous pressure gradient in patients with NASH-related cirrhosis without esophageal varices [319]. The NAVIGATE phase 2b/3 trial (NCT04365868) will assess an 18-month course of belapectin compared to a placebo in NASH patients with compensated cirrhosis, to monitor those who will develop new esophageal varices and clinically significant cirrhosis-related events.

Selonsertib is an oral experimental inhibitor of ASK1 (apoptosis signal-regulating kinase-1). The STELLAR-3 and STELLAR-4 phase 3 trials showed that the use of selonsertib was well tolerated but did not improve liver fibrosis without worsening NASH in patients with stage 3 fibrosis or with compensated cirrhosis [236].

#### 4.3.7. Stearoyl-Coenzyme A Desaturase-1 (SCD1) Inhibitors

Aramchol is an oral modulator of liver stearoyl-coenzyme A desaturase-1 (SCD-1), which is involved in fatty acid biosynthesis, liver steatosis, and fibrosis [320,321]. Aramchol improved liver steatosis by reducing hepatic lipid accumulation (−12.5%) after 3 months of treatment in a phase 2 clinical trial [322].

A systematic review and meta-analysis exploring the effects of aramchol vs. placebo in patients with steatotic liver and including 3 clinical trials, documented any effect of this drug on ALT, AP, glycated hemoglobin, total cholesterol, triglycerides, insulin resistance, and insulin levels [323]. However, in the ARREST phase, 2b double-blind trial, aramchol 600 mg/per day for 52 weeks in patients with overweight or obesity and prediabetes was safe, reduced liver fat by −16.7% in hepatic lipid accumulation compared to only a −5% in the placebo group. NASH and fibrosis improved with a 29.1% decrease in serum ALT and a marked improvement in fibrosis less than 1 grade. It must be noted, however, that the decrease in hepatic lipids was not statistically significant [230]. Aramchol is being compared vs. placebo in the ARMOR phase 3 trial (NCT04104321) enrolling patients with advanced fibrosis and NASH checked for NASH resolution, fibrosis improvement, and clinical outcomes during NASH progression.

#### 4.3.8. DGAT Inhibitors and FASN Inhibitors

Trials are in progress with molecules inhibiting the intrahepatic triglyceride synthesis. The enzyme DGAT consists of DGAT1 and DGAT2 isoforms which have substrate specificities [324] and catalyze the conversion of DAG to triglycerides, a step at the end of triglyceride synthesis. DGAT2 is liver-specific and DGAT2-deficient mice develop reduced hepatic lipid accumulation compared to normal mice [325]. The DGAT2i PF-06865571 reduced liver lipid accumulation in a phase I clinical trial, despite the drug increasing the risk of diarrhea [326]. More data are awaited from another phase II clinical trial (NCT04399538) [327].

The lipogenic enzyme fatty acid synthase (FASN) inhibitors like TVB-2640 (NCT03938246, NCT04906421) are currently under evaluation either alone or in combination with other compounds. In a phase 2 study, patients received a placebo or 25 mg or 50 mg of TVB-2640 orally daily for 12 weeks. Compared to baseline, change in lipid was +4.5%, −9.6%, and −28.1% in the control group, TVB 25 mg and TVB 50 mg, respectively. Additional effects were decreased ALT and LDL levels, in a dose- and time-dependent fashion. The small sample size is one limitation of the study [328].

#### 4.3.9. MGAT2 Inhibitors

MGAT2 is overexpressed in the liver and small intestine [329,330]. Selective MGAT2 inhibition can decrease gut TG synthesis, delay fat absorption, and decrease the risk of diarrhea while improving liver steatosis via weight loss. BMS-963,272 is a novel selective MGAT2 inhibitor that in mice with MASH improved liver inflammation and fibrosis without diarrhea. In a phase 1 trial, the use of BMS-963,272 was associated with decreased body weight, increased GLP-1 and PYY levels, and no adverse effects [331].

#### 4.3.10. Dimethyl Peptidase 4 (DPP4) Inhibitors 

The DPP4 is expressed on several cell surfaces and functions to cleave different substrates. One target is the GLP-1, and this step has a regulatory effect on diabetes [332]. Notably, individuals with MASLD have increased DPP4 levels along with hepatocyte apoptosis and fibrosis [333]. MASH inflammation and fibrosis improve in mice treated with DPP4 inhibitors [334]. One hypothesis was therefore that the inhibition of DPP4 activity can increase GLP-1 activity, with beneficial effects on MASLD. However, the DPP inhibitor sitagliptin failed to reduce hepatic lipid accumulation and NAS score in a phase 2 trial [335].

#### 4.3.11. Ketohexokinase (KHK) Inhibitors

KHK, the rate-limiting enzyme in fructose metabolism, is responsible for the conversion of fructose to fructose 1-phosphate. Excessive fructose intake is a risk factor for MASLD [336,337,338], and is associated with increased hexokinase levels, deranged fatty acid oxidation, and enhanced DNL. In this context, the hepatic steatosis and insulin signal transduction worsen [339]. Knocking out the liver hexokinase appears to decrease the fructose-induced hepatic damage [340]. PF-06835919 and hexokinase inhibitors in clinical trials decreased hepatic lipid accumulation, despite insulin resistance did not improve [302]. Other trials are awaited. 

#### 4.3.12. Miscellanea

Icosabutate, a synthetic omega-3 fatty acid, is an eicosapentaenoic acid that resists oxidation and does not undergo liver accumulation. This molecule might protect against hepatic oxidative stress, inflammation, and fibrosis. In the ICONA phase 2b study (NCT04052516), different doses of icosabutate are used to assess the efficacy of the resolution of MASH (i.e., the disappearance of ballooning with lobular inflammation) without worsening of fibrosis. 

Mitochondria are important players during steatosis and can become potential therapeutic targets as part of mitochondrial therapy [41]. The thiazolidinedione MSDC-0602 K modulates the mitochondrial pyruvate carrier (mPC), a protein complex governing the entry of pyruvate into the mitochondria [122]. In patients with biopsy-proven MASH and stage 1–3 fibrosis, the EMMINENCE phase 2b placebo-controlled randomized trial reported a dose-dependent improvement in the glycemic control and liver enzymes but failed to meet the histological outcomes, i.e., ≥2-point NAS improvement without worsening fibrosis, MASH resolution, and fibrosis reduction [341]. 

PXL065 is a deuterium-stabilized R-pioglitazone lacking PPAR-γ activity. PXL065 has non-genomic target activities via mitochondrial pyruvate carrier and acyl- CoA synthetase 4 inhibition and is being tested in a phase 2 trial in non-cirrhotic patients with NASH (NCT04321343). Preliminary analyses suggest that PXL065 reduces liver fat content in about 40%, and improves at least 1 fibrosis stage in about 30–50% of patients. About 30% of patients showed NASH resolution after 36 weeks of treatment with good safety. Side effects were minimal since PXL065 lacks the PPAR-γ activity of glitazones [342].

Policaptil Gel Retard (PGR) is a natural macromolecular complex covered by a European patent no. 1679009. PGR polysaccharides can reduce carbohydrate and fat absorption rates. PGR was successfully used in adolescents and adults with metabolic syndrome and T2DM and successfully reduced circulating levels of insulin, lipids, and post-prandial triglycerides. The improvement was also evident in insulin resistance and body fat distribution [343,344]. A non-inferiority effect of PGR compared to metformin was also evident in obese adults with metabolic syndrome and T2DM [345]. A recent spontaneous, longitudinal, single-blind, randomized clinical study enrolled 245 individuals with metabolic syndrome and T2DM and randomized to PGR or placebo for 24 weeks when a low-calorie diet and intensified physical activity were allowed. PGR added to lifestyle changes improved lipid and glucose metabolism-related parameters, including insulin resistance, and significantly reduced not only visceral fat but also liver fat content and related liver fibrosis severity. The effect of PGR was likely related to a reduction in the post-meal blood glucose and insulin peaks [346].

The potential role of microbiota manipulation as a therapeutic approach to MASLD must be also considered. Gut microbiota is likely involved in the pathogenesis of MASLD due to reduced bacterial diversity, altered *Firmicutes/Bacteroidetes* ratio, and a relative abundance of alcohol-producing bacteria [57]. Correction of gut dysbiosis can oppose the disrupted intestinal barrier and hyperpermeability, the flow of bacterial products (i.e., lipopolysaccharides), immune system and inflammatory activation in the intestine, endothelial barrier, in the liver, and at a systemic level [56,57,294]. The close link between overweight/obesity and the steatotic liver opens the venue to potential novel approaches by targeting gut microbiota [55]. For example, a randomized, double-blind, placebo-controlled pilot study with 26 diabesity patients was conducted. Patients received for 6 months a daily dose of a multispecies synbiotic containing 1.5 × 10^10^ CFU of a blend *B. bifidum W23, B. lactis W51, B. lactis W52, L. acidophilus W37, L. casei W56, L. brevis W63, L. salivarius W24, Lc. lactis W58, Lc. lactis W19* plus galacto-, fructo-oligosaccharides, glucomannan, minerals and D3-B2 vitamins. Results showed significant beneficial effects on hip circumference, serum zonulin, and overall quality of life [347]. Further studies are in progress to extend such effects in patients with MASLD/MASH.

There is a rationale to consider the potential benefits of fecal microbiota transplantation (FMT) in MASLD, since the concept of gut–liver axis includes the gut microbiota which appears to be involved in the development of hepatic steatosis [56,57,294]. The gut microbiota may differ between MASLD patients and healthy people [57], and in a phase 2 randomized clinical trial, 21 MASLD patients underwent endoscopic allogeneic or autologous fecal transplantation. Although FMT reduced small intestinal permeability in MASLD, it did not improve insulin resistance or hepatic PDFF lipid accumulation after six months [348]. Interestingly, a single dose injection of *A. soehngenii* to the duodenum in patients with metabolic syndrome showed robust GLP-1 production and peripheral glycemic homeostasis [349,350]. 

## 5. Conclusions and Perspectives

**Figure 8:**
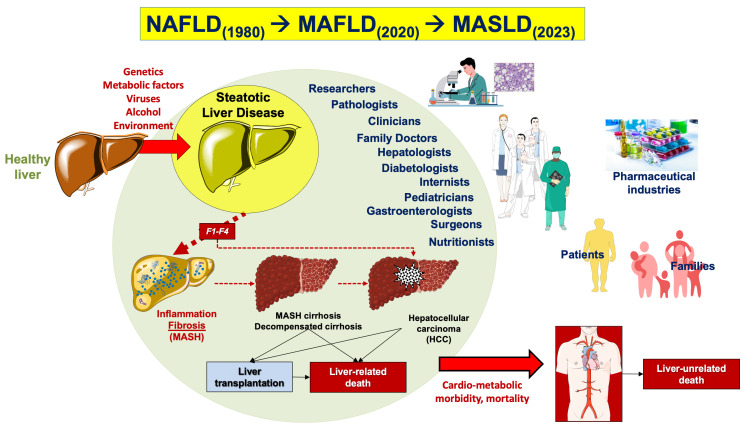
The process showing the change of nomenclature for liver steatosis (from 1980 to 2023), in relation to the progression of disease and interplay between several professionals and stakeholders. The terminological evolution (i.e., from NAFLD to MAFLD and, finally, to MASLD) has been paralleled by a progressive growth of knowledge about the combined effects of diverse pathogenic factors (i.e., genetic and external factors) in the onset and progression of steatotic liver disease. This evidence, in particular, underscores the association of fat overstorage in the liver not only with the possible progression of hepatic damage but also with systemic metabolic disturbances and cardiovascular risk factors, and the need for a multi-disciplinary and transversal approach to this disease. The cooperation between different stakeholders (including subjects at risk, health professionals, and pharmaceutical industries) could significantly improve either the management of disease and the implementation of primary and secondary prevention measures.

Since 1980, researchers have been focusing on pathophysiological, diagnostic, and therapeutic aspects of NAFLD because of the risk of progression to severe chronic liver diseases. Since then, NAFLD has become the most frequent chronic liver disease and pathogenic mechanisms have been partly unveiled. Starting from this last evidence, between 2020 and 2023 a new nomenclature appeared in the scientific literature, i.e., MAFLD and MASLD, respectively. Despite a still ongoing debate on the final acronym, the robust association of NAFLD/MAFLD/MASLD with metabolic disturbances and cardiovascular risk factors has been globally acknowledged, and risk assessment and effective management of patients with this disorder will require close collaboration between multiple stakeholders of the medical professional community (Figure 8). The high healthcare burden associated with MASLD makes the search for new, effective, and safe drugs a major pressing need while it remains a challenging quest. A unique drug to address all key aspects of MASLD is still missing and is quite unlikely to be found, since MASLD is a disease with complex pathogenesis and highly heterogeneous clinical outcomes, including liver-specific and systemic implications. Clinical trials with single drugs or their limited combinations demonstrate that even the best results are invariably achieved in no more than about half of the treated patients. This experience suggests that lasting success in the management of MASLD will only result from the combination of lifestyle modification and pharmacotherapy targeting multiple molecular targets and taking the genetic predisposition and susceptibilities of individual patients into account. Recent and promising advances indicate that we may soon enter the era of precise and personalized therapy for MASLD/MASH.

## Figures and Tables

**Table 1:** Phase II, III, and IV clinical trials for (N)MASH treatment.

Trial Phases	ClinicalTrials.gov	Start Date of Trial (Year-Month-Day)	Drug	Molecular Mechanism (Target)	Patients	Main Findings	Adverse Effect	References
II	NCT02970942	2016-11-30	Semaglutide	Agonist GLP1	320 NASH patients	NASH resolution without fibrosis improvement	Nausea, diarrhea, abdominal discomfort, reaction site injections	[226]
II	NCT01237119	2010-08	Liraglutide	Agonist GLP1	52 NASH, NAFLD, Obese, and T2DM patients	NASH resolution but with worsening of fibrosis	Nausea, diarrhea, abdominal discomfort, reaction site injections	[227]
II	NCT03008070	2017-02-07	Lanifbranor	Pan-PPAR agonist	247 NASH patients having stable T2DM	Improvement of NASH and fibrosis per liver biopsy	Nausea, diarrhea, peripheral edema, anemia, and weight gain	[228]
II	NCT03350165	2017-12-27	Pemafbrate	PPARα agonist	118 NASH and NAFLD patients	No differences were observed in liver fat content but a reduction in stiffness was achieved	Mild-or-moderate adverse events	[229]
II	NCT02279524	2015-04-29	Aramchol	Partial inhibitor of hepatic stearoyl-CoA desaturase	247 Obese, T2DM diagnosed with NASH	No changes in liver fat but there was an improvement in liver fibrosis by ≥1 without worsening of NASH on liver histology	Well tolerated	[230]
II	NCT04929483	2021-06-04	Pegozafermin	FGFR1,2,3,Stimulants	222 diagnosed with NASH	improvements in fibrosis	nausea and diarrhea.	[231]
II	NCT03976401	2019-05-28	Efruxifermin	FGF21R Agonist	110 NASH, NAFLD Patients	Generally safe; significantly reduced liver fat	Gastrointestinal abnormalities, pulmonary embolism, acute pancreatitis with subsequent diabetic ketoacidosis	[232]
II	NCT02854605	2016-10-26	Cilofexor tromethamine, GS-9674	FXR Agonists, insulin sensitizers	140 NAFLD-diagnosed patients	significant reductions in hepatic steatosis, liver biochemistry, and serum bile acids in NASH patients	Vertigo, abdominal pain, diarrhea, fatigue, pruritus, headache	[233]
III	NCT00063622	2005-01	Pioglitazone/vitamin E	PPARγ agonist	247 NASH patients	Pioglitazone was effective	Weight gain	[234]
III	NCT03061721	2017-04-06	Saroglitazar	Dual PPARα and PPARγ agonist	106 NAFLD patients	significantly improved all of ALT, LFC, insulin resistance, and atherogenic dyslipidemia	Diarrhea and cough	[235]
III	NCT03053050	2017-02-13	Selonsertib	ASK1 Inhibitors	808 NASH patients	no anti-fibrotic effect	Back pain, pruritus, cough, nasopharyngitis, cirrhosis, Diarrhoea.	[236]
III	NCT00267670	2005-03	Pentoxifylline	PDE inhibitors, TNF-α inhibitor	26 NASH Patients	improvement in serum aminotransferases as well as some histological features of NASH when compared to baseline measurements, no effect on fibrosis	Headache and abdominal cramps	[237]
III	NCT03028740	2017-04-05	Cenicriviroc	CCR2 antagonists, CCR5 antagonists	1778 NASH patients	Safe, and there was an improvement in fibrosis, no worsening of steatohepatitis	Nausea, Diarrhoea, abdominal pain, fatigue, arthralgia, headache	[238]
III	NCT04104321	2019-09-23	Aramchol	SCD1 inhibitors	150 diagnosed with NASH	Suspended		Suspended
IV	NCT00227110	2002-10	Pioglitazone	PPARγ Agonist	55 NASH patients	metabolic and histologic improvement in subjects with non-alcoholic steatohepatitis	Fatigue and mild lower-extremity edema	[239]

Legend: ALN, Alnylam; ALT, Alanine Transaminase; ASK-1, Apoptosis Signal-Regulating Kinase 1; CCR2, Chemokine Receptor 2; CCR5, Chemokine Receptor 5; CystLT, Cysteinyl Leukotriene Receptor; FGF21R, Fibroblast Growth Factor 21 Receptor; FGFR, Fibroblast Growth Factor Receptor; FXR, Farnesoid X Receptor; GPD1, Glycerol-3-Phosphate Dehydrogenase; GLP1, Glucagon-Like Peptide-1; GR, Glucocorticoid Receptor; HSD13, Hydroxysteroid Dehydrogenase 13; LFC, Liver Fat Content; MRI, Magnetic Resonance Imaging; MR, Mineralocorticoid Receptor; NAFLD, Non-Alcoholic Fatty Liver Disease; NASH, Non-Alcoholic Steatohepatitis; PDFF, Proton Density Fat-Fraction; PDE, Phosphodiesterase Inhibitors; PPAR-α, Peroxisome Proliferator Activated Receptor Alpha; PPAR-γ, Peroxisome Proliferator-Activated Receptor Gamma; PRKAB1, Protein Kinase, AMP-Activated Beta 1; RNAP, RNA Polymerase; SCD-1, Stearoyl-CoA Desaturase Enzyme 1; SGLT2, Sodium-Glucose Co-Transporter-2; THR-β, Thyroid Hormone Receptor-Beta; TNF-α, Tumor Necrosis Factor Alpha; T2DM, Type 2 Diabetes Mellitus.

## Data Availability

Not applicable.
